# Characteristics of dorsal root ganglia neurons sensitive to Substance P

**DOI:** 10.1186/1744-8069-10-73

**Published:** 2014-11-27

**Authors:** Eder Ricardo Moraes, Christopher Kushmerick, Ligia Araujo Naves

**Affiliations:** Departamento de Fisiologia e Biofísica ICB, Universidade Federal de Minas Gerais, Belo Horizonte, MG 31270-901 Brasil

**Keywords:** Dorsal root ganglia, Substance P, Acid sensing ion channels, P2X channels, TRPV1 channel

## Abstract

**Background:**

Substance P modulates ion channels and the excitability of sensory neurons in pain pathways. Within the heterogeneous population of Dorsal Root Ganglia (DRG) primary sensory neurons, the properties of cells that are sensitive to Substance P are poorly characterized. To define this population better, dissociated rat DRG neurons were tested for their responsiveness to capsaicin, ATP and acid. Responses to ATP were classified according to the kinetics of current activation and desensitization. The same cells were then tested for modulation of action potential firing by Substance P.

**Results:**

Acid and capsaicin currents were more frequently encountered in the largest diameter neurons. P2X3-like ATP currents were concentrated in small diameter neurons. Substance P modulated the excitability in 20 of 72 cells tested (28%). Of the Substance P sensitive cells, 10 exhibited an increase in excitability and 10 exhibited a decrease in excitability. There was no significant correlation between sensitivity to capsaicin and to Substance P. Excitatory effects of Substance P were strongly associated with cells that had large diameters, fired APs with large overshoots and slowly decaying after hyperpolarizations, and expressed acid currents at pH 7. No neurons that were excited by Substance P presented P2X3-like currents. In contrast, neurons that exhibited inhibitory effects of Substance P fired action potentials with rapidly decaying after hyperpolarizations.

**Conclusion:**

We conclude that excitatory effects of Substance P are restricted to a specific neuronal subpopulation with limited expression of putative nociceptive markers.

## Background

Dorsal root ganglia (DRG) neurons are primary sensitive neurons that transduce and convey sensory information to the central nervous system. The DRG neuronal population is highly heterogeneous in terms of size and molecular constitution, and the classification into cell subpopulations has provided basis to understanding primary sensorial neuronal function (for a critical review see [1]). Cell size was one of the earliest criteria used to differentiate DRG neurons, as the diameter of perikarya range from 14 to 75 μm [2]. It has been demonstrated by both light and electronic microscopy that there are two populations of DRG neurons which can be distinguished based on their cytoplasmic appearance: small dark neurons and large light neurons [3]. Frequency distribution histograms of cell sizes also show at least two populations [2, 3]. Pain sensation is usually considered to be conveyed to central nervous system by neurons with small perikarya [4] although some techniques have identified both small (≤30 μm) and large (≥40 μm) nociceptors [5].

One subpopulation of small DRG neurons produces Substance P, a neuropeptide that is released at regions in the CNS associated with transmission of pain including laminas I, II and V of the dorsal horn [6]. Substance P is also released by DRG neuron projections to the periphery, where it contributes to neurogenic inflammation in many tissues [7].

Aside from the effects of Substance P on second order sensory neurons [8, 9] and peripheral organs [7], Substance P also affects some primary sensory neurons [10–18]. At this site, Substance P changes the excitability of a neuronal subpopulation [11, 13, 14, 17] and modulates nociceptive stimulus transducing molecules including the TRPV1 [16], P2X3 purinergic channels [15] and acid sensing ion channels [18], also in a subset of neurons. It is presently unknown if the actions of Substance P are restricted to the neurons that expresses these molecules. In this study, we recorded currents produced by ATP, acid and capsaicin in the same neurons that we then evaluated for changes in excitability produced by Substance P. We studied the size distributions of the neurons that respond to these stimuli and also correlated sensitivity to Substance P with cell size, action potential waveform parameters and the presence of ligand gated currents.

We find that Substance P increases the excitability of some cells and inhibits others. Neurons that are excited by Substance P are large, express ASIC currents at pH 7 and do not express P2X3-like ATP currents. They present action potentials with a large overshoot and long after hyperpolarization. Neurons that are inhibited by Substance P are largely indistinguishable from the general population, except for the presence of a relatively fast decaying after hyperpolarization.

## Results

### The effect of substance P on excitability

**Figure 1:**
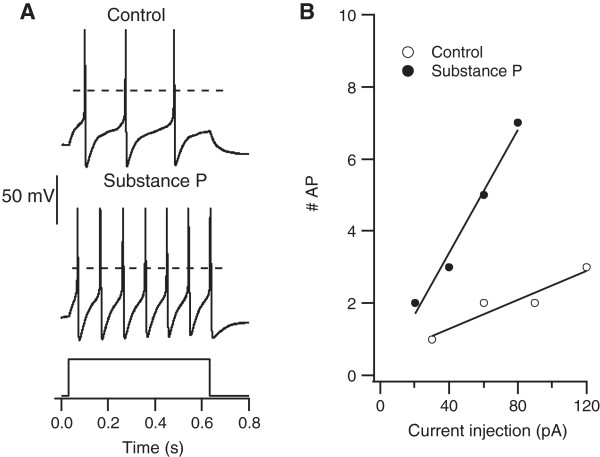
**Substance P changes the excitability of a sub-population of DRG neurons. A**. Action potentials generated by current injection in a DRG neuron before (*upper trace*) and during treatment with 2 μM Substance P (*lower trace*). The *broken line* indicates 0 mV. Resting potential was -55 mV in control and -50 mV in Substance P. Current injection was 120 pA in control and 80 pA in Substance P. **B**. Input-output relationship for the same cell when stimulated with 1-4 times its threshold current (control: 30 pA, Subtance P: 20 pA).

It has been shown that Substance P increases the number of AP discharges in a subset of DRG neurons [11, 13], but the properties of Substance P sensitive neurons are poorly defined. To learn more about the characteristics of these neurons, we used the effect of Substance P on Action potential (AP) firing to identify SP-sensitive cells. Figure 1A shows a representative recording of APs in a Substance P sensitive neuron before (control) and after treatment with 2 μM Substance P. To quantify effects of Substance P, the relationship between current injection and number of action potentials was measured when stimulating at 1 to 4 times the current threshold (Figure 1B, see Methods). Of 72 cells tested, 20 were found to be sensitive to Substance P. In these 20 cells, Substance P produced excitatory effect in 10 cells, while it decreased excitability in the other 10 cells.

### Action potential properties of neurons sensitive to substance P

Some types of DRG neurons, including nociceptors, fire broad action potentials with large overshoots and long duration after hyperpolarizations (AHP) [19, 20]. To test if the neurons sensitive to Substance P share any of these properties, we examined cells that were sensitive to Substance P compared to the remaining population. As shown in Table 1, the width of action potentials recorded in cells that responded to Substance P was not different from the non responding cells. Cells that increased their excitability in the presence of Substance P had substantially larger overshoots and longer AHP half-widths compared to the rest of the population. In contrast, cells that were inhibited by Substance P had significantly shorter AHP half-widths (Table 1). We conclude that the action potential properties of neurons excited by Substance P share some properties previously described for nociceptors [19, 20].

**Table 1:** **Size and action potential (AP) properties of neurons sensitive to substance P**

Property		Whole population	Substance P sensitive		
			All	Excite	Inhibit
		(n = 72)	(n = 20)	(n = 10)	(n = 10)
**Capacitance (pF)**	Mean	61.5	76	82.2	69.8
	SD	30.6	32.7	29.1	36.4
	P	---	0.02	0.03	0.38
**AHP Half-Width (ms)**	Mean	18	20	27	10
	SD	15	12	11	6
	P	---	0.37	0.01	0.004
**AP Width at 0 mV (ms)**	Mean	2.6	2.9	3.3	2.5
	SD	1.81	1.79	1.39	2.16
	P	---	0.52	0.18	0.79
**Resting Potential (mV)**	Mean	−60	−61	−62	−60
	SD	9.4	9.2	6.9	11.3
	P	---	0.28	0.19	0.74
**AP Overshoot (mV)**	Mean	51	53	63	43
	SD	17	18	13	18
	p	---	0.38	0.005	0.24

Values for p were calculated by t test comparing each group with the remaining cells from the whole population. AHP, Afterhyperpolarization.

### Correlations between neuronal size and sensitivity to acid, ATP, capsaicin and substance P

Aside from action potential properties, another criterion used to distinguish different types of DRG neurons is the soma size. For example, nociceptors are often associated with small diameter neurons [2, 21]. As shown in Table 1, neurons that respond to Substance P are among the largest in our populations and are significantly larger than the rest of the population.

**Figure 2:**
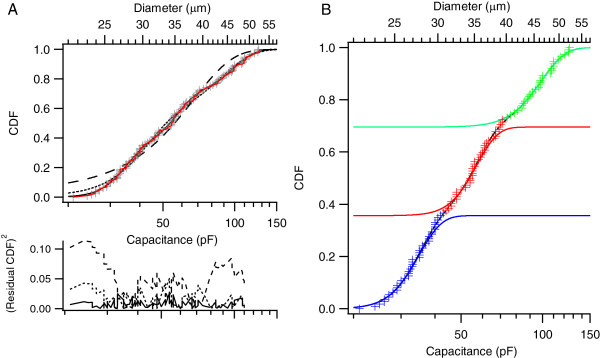
**The size distribution of DRG neurons are best described by three populations. A**. The cumulative distribution function of cell sizes was fit by one, two, or three normal populations (broken-line, dotted line and solid line, respectively). Analysis of the residual (lower traces) indicated a significantly better fit with three populations compared to one or two populations. **B**. The three cell size populations identified in A: small cells (Blue) with capacitance <44 pF (diameters up to 30 μm), large cells (green) with capacitance >74 pF (diameter > 40 μm), and intermediate sized cells (red).

Previous studies have divided DRG neurons into multiple populations based on cell size [2, 3]. Figure 2 shows the cumulative distribution of soma sizes of 113 cells recorded in this study. Based on the goodness-of-fit as well as model selection criteria (see Methods) we found that three normal populations provided the best fit to our data with means of 34 pF (27 μm, 36% of cells), 55 pF (35 μm, 34% of cells) and 95 pF (46 μm, 30% of cells). We shall refer to these groups as small, medium, and large neurons.

**Figure 3:**
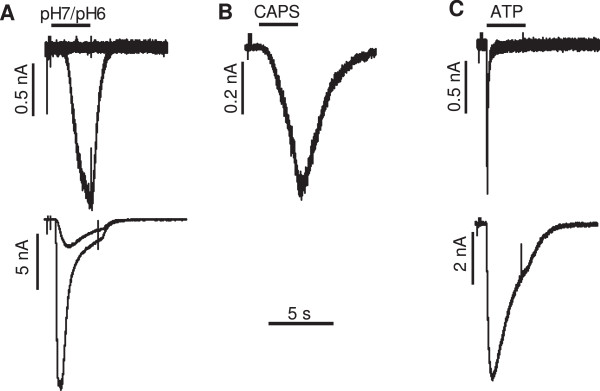
**Representative currents generated by A) acid (pH 7 or 6.0), B) 3** **μM capsaicin (CAPS) or C) 50** **μm ATP.** The black bars on the top of the currents indicate the times during which the substances were applied.

In order to determine the size distribution of neurons that present TRPV1, ASIC and P2X channels, we measured currents evoked by capsaicin (3 μM), low pH (pH 7.0 and pH 6.0), and ATP (50 μM). Figure 3 shows representative currents obtained for each of these three ligands. Based on their activation and desensitization kinetics [22] we classified the ATP evoked currents as P2X3-like or non P2X3-like. (Figure 3 C, top).

**Figure 4:**
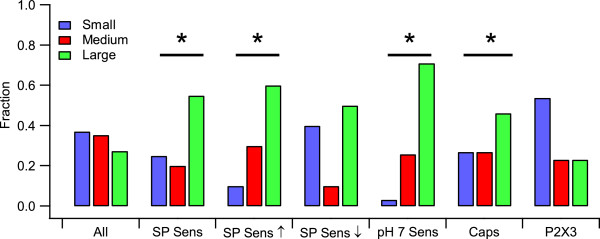
**Substance P sensitivity is more prevalent in large neurons.** All cells were tested for sensitivity to Substance P after testing for expression of currents elicited by pH7, capsaicin and ATP. ATP currents were classified as P2X3 based on their kinetics of activation and deactivation. The fraction of small (blue), medium (red), or large (green) cells for each group was determined for all cells, all Substance P sensitive cells (SP Sens), cells that were excited by Substance P (SP Sens ↑), cells that were inhibited by Substance P (SP Sens ↓), all cells that expressed ASIC channels (pH 7 Sens), all cells that expressed capsaicin receptors (CAPS), and all cells that expressed P2X3-like ATP currents (P2X3). Bars marked with the symbol * show groups whose size distribution is statistically different from all cells outside that population (p < 0.05, Fisher’s exact test).

The size distribution of Substance P sensitive neurons and neurons sensitive to acid, capsaicin and ATP is shown in Figure 4. Substance P sensitive neurons, and especially those with an excitatory response to Substance P, were more common among large diameter neurons. A similar pattern was observed for sensitivity to pH 7, which was much more frequently observed in large neurons than medium or small neurons. Capsaicin sensitivity was also more frequent in large neurons, although the highest density of capsaicin current was observed in small neurons (not shown). In contrast, ATP evoked P2X3-like currents occurred predominantly in small neurons. The distributions of P2Xnon3-like currents in small, medium and large neurons were not significantly different (data not shown).

### Correlations between responsiveness to Substance P and ligand gated channels

**Figure 5:**
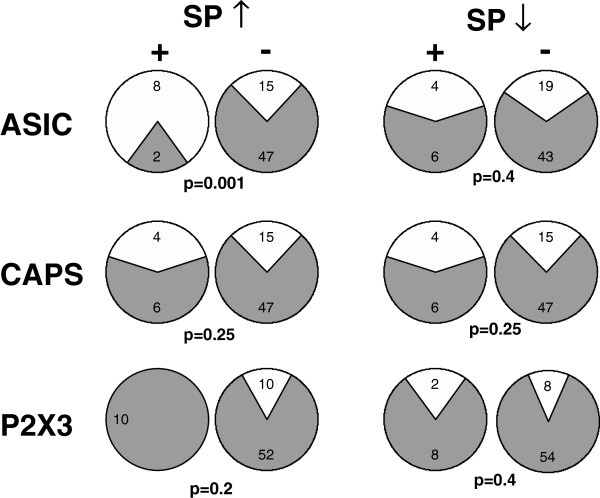
**Excitation by Substance P correlates with expression of ASIC channels.** The number of cells that expressed (white) or did not express (gray) each of the channels listed on the left was determined for cells that were (+) or were not (-) excited (↑) or inhibited (↓) by Substance P. Statistical p values for the differences in distributions were calculated using Fisher’s exact test and are given below each pair of pie charts.

We measured the frequency of expression of ASIC currents at pH 7, capsaicin currents and P2X3-like ATP currents in SP-sensitive and SP-insensitive neurons. As shown in Figure 5, most cells (80%) that responded to Substance P with an increase in excitability also presented ASIC3-like acid currents at pH 7, compared to only 24% in the remaining population. This difference was statistically significant (p = 0.001). The correlation between responsiveness to substance P and acid currents was not observed for cells that were inhibited by Substance P. Capsaicin currents were found in cells of all sizes, but we observed no correlation between expression of capsaicin currents and sensitivity to substance P. None of the 10 cells that increased excitability due to Substance P application presented P2X3 current, whereas no such negative correlation was found among cells inhibited by Substance P.

## Discussion

### Relations between neuronal size and sensitivity to acid, capsaicin, ATP and substance P

DRG neurons are remarkably diverse in terms of their cell body diameter [2, 3], and several studies have attempted to associate cell size with function [23–25]. The observations that nociceptive neurons have low conduction velocities [21] (but see [26]), and that cell size and conduction velocity are correlated for Aδ and C fiber cells [3], led to the concept that nociceptors have small cell bodies.

The membrane receptors P2X3, ASIC3 and TRPV1 have been associated with nociceptive stimuli transduction [21, 23, 27, 28]. In accordance with its nociceptive function, we found that expression of a P2X3-type current was most frequently seen in small neurons. In contrast, sensitivity to capsaicin was detected in neurons of all sizes, with a significantly higher frequency in larger neurons. This observation conflicts with previous studies that found TRPV1 imunoreactivity and mRNA expression was concentrated in small and medium diameter neurons [29, 30]. One possible explanation for this discrepancy is that although we observed a higher frequency of cells that expressed capsaicin receptors in large cells, the capsaicin current density was highest in small cells (not shown). Such high expression densities are more likely to give significant labeling. Moreover, in the studies that correlate neuronal size and capsaicin currents, there is little [31] or no [32] sampling of neurons larger than about 40 μm/70 pF, which would exclude the cells we call large. It thus appears that the frequency of capsaicin currents in the largest of DRG neurons may be underestimated. Regarding to acid responding neurons, we found that these currents were much more prevalent in large neurons. Although there are reports that ASICs are localized to small neurons [4, 25, 33], others have found that ASICs are present predominantly in large neurons [34, 35].

We observed a higher frequency of responses to Substance P in large diameter neurons. Substance P receptor (NK1) mRNA has been found to be present mostly in small DRG neurons [10]. Perhaps for this reason, in many studies the effects of Substance P were tested exclusively in small DRG cells [12, 16, 17]. Nonetheless, our data agrees with the finding that NK1 receptors, at the plasma membrane, are localized especially in intermediate and large DRG neurons [36]. Because substance P is produced primarily in small neurons [6], the effects we observe are unlikely to be mediated by autoreceptors.

### Substance P activation targets multiple ion channels

It has been shown that substance P modulates ligand-gated channels including P2X3 ATP receptors [15], TRPV1 capsaicin receptors [16] and ASIC3 channels [18], as well as several types of voltage-gated channels [11–14, 17, 37, 38]. In the present study, we classified neurons as SP-sensitive based on the effect of substance P on action potentials evoked by current injection. It is thus likely that the effects of SP we observed are due to modulation of voltage-gated channels.

In half of the cells that were sensitive to Substance P, the effect was a decrease in excitation. Previous studies indicate several potential inhibitory mechanisms of Substance P. It has been shown that Substance P activates a potassium channel leading to hyperpolarization and blocks a hyperpolarization-activated Ih current, and these two effects can act synergically to decrease excitability [37, 38]. The actions of Substance P on these two channel types could account for the reported activation of an outward current in 23% of the cells responsive to SP [11]. Another possible cause of decreased excitability by SP is a block of calcium channels observed in some neurons [12].

Regarding to the excitatory effects, several reports have shown that Substance P increases the firing rate of subsets of DRG neurons [11, 13, 14, 17]. Mechanisms that have been demonstrated that could explain this action include block of inactivating potassium currents [14] and potentiation of tetrodotoxin-resistant sodium channels [17].

Our study demonstrates that excitatory actions of Substance P are most commonly encountered in neurons that express ASICs. Several studies have shown that the types of ion channels expressed by DRG neurons can vary within different subpopulations [39–42]. It remains to be determined if the expression of ASICs is associated with the expression of ion channels modulated by substance P.

### Possible implications of the excitatory effect of Substance P

One of our major findings is that Substance P excites predominantly cells with ASIC-like currents but not neurons with P2X3-like currents. These P2X3 positive neurons are exclusively non-peptidergic [43], and the lack of an excitatory effect of Substance P in these neurons suggests an additional criterion to separate this subpopulation. ASICs have been implicated in the transductions of nociception and mechanosensation [44–46]. It is thus possible that Substance P modulates pain and mechanoreception. Supporting such a modulatory role on pain is the reported antinociceptive effect of Substance P in a model of muscle acid-induced hyperalgesia [18]. In this study, the authors propose a model of a negative feed-back control of ASIC activity at the nerve ending mediated by substance P release.

ASICs have been described in afferents from skeletal muscle [47], skin [48], heart [27] and other viscera [49, 50]. At these sites, ASICs serves as sensors that triggers homeostatic responses such as pressor reflex, vasodilation, control of digestion, behavior towards food intake, as well as pathological reflexes and pain. If substance P can be released by ASIC activation, also at these sites, it may control some of these functions.

Petruska et al. [4] have subclassified 9 types of DRG neurons, based on their current responses to three voltage protocols, and they have looked at their size, action potential properties and responses to protons, capsaicin and ATP. The cells that they classified as type 7 are very sensitive to acid and have long after hyperpolarization. Nonetheless, these cells were very small, so they likely represent a different population from the neurons we found responsive to Substance P.

Molliver at al. [51] demonstrated the existence of ASIC3 positive and P2X3 negative thick caliber axons that project to muscle blood vessels. Some of these neurons express TRPV1 and most of them produce calcitonin gene related peptide, which is usually co-localized with substance P [52]. The authors suggested that these afferents may be muscle metaboreceptors, neurons that sense the metabolic state of the muscle and can trigger reflexes and pain in response to stress. Interestingly, muscle metaboreceptors have a large diameter [53] and fire broad action potentials [47]. Thus, these neurons share many of the properties we describe for the SP positive cells. It is thus possible that the neurons we found to be sensitive to Substance P are metaboreceptors. Molliver et al. [51] also demonstrated large ASIC3 positive neurons with a pattern of non-nociceptive, non-propioceptive mechanoceptors, so the neurons sensitive to Substance P could be mechanoceptors. It has been demonstrated that ASIC3 are the sensors in mechanoceptors involved in cutaneous vasodilation produced by pressure [54], a reflex that is very important for skin ulcer prevention. If Substance P acts on these metabo and mechano receptors it could modulate these reflexes. These hypotheses remain to be tested.

## Methods

All experiments were carried out in accordance with the National Institute of Health Guide for the Care and Use of Laboratory Animals using protocols approved by the institutional Ethics Committee for the Use of Animals, Universidade Federal de Minas Gerais (CEUA-UFMG).

### Cell isolation

Neurons were isolated from dorsal root ganglia with minor modifications of the protocol described by Eckert et al. [55]. Briefly, male adults Wistar rats were killed by decapitation. Ganglia from all spinal segments were removed and placed in cold Ca2 + -free Ringer solution. Ganglia were sectioned with iridectomy scissors and incubated at 37°C for 20 minutes in Ca2 + -free Ringer plus papain (1 mg/ml) activated by cystein (0.03 mg/ml), followed by 20 minutes in Ca2 + -free Ringer plus collagenase (2.5 mg/ml), also at 37°C. The enzyme incubation was stopped with F-12 media supplemented with 10% fetal bovine serum (FBS) and 100 U/ml penicillin/streptomycin. The cells were released by gentle trituration through Pasteur pipettes with fire-polished tips. Cells were platted on glass discs pre-coated with poly-D-lysine and laminin inside Petri dishes. After 2 hours in an incubator at 37°C to allow the cells to settle, the media was exchanged with Leibovitz’s L-15 medium supplemented with 10% FBS, 5 mM glucose, 5 mM NaHEPES and 100 U/ml penicillin/streptomycin at room temperature. In about half of the experiments, 50 ng/ml NGF was add to the L-15 media. We observed no significant difference in any parameter from cells treated with NGF compared with untreated cells and the data from both groups are merged.

### Electrophysiological recordings

Ligand gated currents and action potentials from DRG neurons were recorded 12-36 hours after plating using the whole-cell configuration of the patch-clamp method [56] at room temperature (22–24°C). Action potentials were recorded using the current clamp mode. Internal solution consisted of (in mM): K gluconate 30, KCl 30, NaCl 4, MgCl2 5, EGTA 11, HEPES 10, MgATP 2, NaGTP 0.3, pH 7.0. External solution consisted of (in mM): NaCl 140, KCl 2.5, CaCl2 2, MgCl 1, HEPES 10, MES 10, Glucose 7.5, pH 7.4. Currents and action potentials were recorded using a patch clamp amplifier (Axopatch 200B, Axon Instruments, USA), controlled by pClamp7 software (Axon Instruments, USA). Pipettes with tip resistances of 1–2 MOhm were fabricated from capillary (Patch Glass, PG150T, Warner Instrument) utilizing a two stage vertical pipette puller (PP 830 Narishige, Tokyo, Japan), and polished in a microforge (MF 830 Narishige, Tokyo, Japan). The taper of the recording micropipettes was covered with dental wax to approximately 0.1 mm of their tip to reduce their electric capacitance and thus facilitate cancellation of pipette capacitive currents. Holding potential was -70 mV. To evoke ligand-gated currents, an array of eight 10 μl pipettes with their flows controlled by computer driven solenoid valves provided rapid solution exchange. Signals were low-passed filtered at 5 KHz, and sampled at 50 kHz (action potentials) or 10 kHz (ligand gated currents). Acid currents were evoked by rapid exchange of the external solution to pH 7.0 or 6.0. ATP currents were evoked by 50 μM ATP and capsaicin currents by 3 μM capsaicin. The concentrations of agonists used correspond to about 5 times the half maximal effective concentrations of ATP [57] and capsaicin [58]. These concentrations were chosen to ensure full activation of TRPV1 and P2X channels. Neurons were considered to respond to a given stimulus when they generated peak current greater than 100 pA.

### Data analysis

Sensitivity to Substance P was determined by its effect on action potential firing. Threshold current was determined by injecting current in steps of 20 pA. The first such injection that elicited at least one action potential was taken as current threshold. Once threshold was determined, the cell was stimulated at 1, 2, 3, and 4 times its threshold, and the number of action potentials was recorded. This procedure was carried out immediately before treatment with Substance P, and repeated after two minutes treatment with and in the continued presence of Substance P. The data for each cell were fit by a line. For each cell, we recorded the change in the threshold current, number of action potentials generated at control threshold, and the slope of the number of APs *versus* current injection. To test the statistical significance of the changes that occurred, we recorded from 12 cells that received sham Substance P treatment. These cells were treated identically to the experimental group, except that Substance P was omitted from the perfusate. Cells in the experimental group were classified as sensitive to Substance P if the changes in any of the parameters above were outside the mean ±2.2 times the standard deviation of the sham group, which corresponds to p < 0.05.

To separate DRG neurons into multiple populations, we attempted to fit the data with a single normal distribution or the sum of two or more normal distributions. As expected, as the number of distributions increased the goodness of fit improved. We used the Akaike Information Criteria to determine if the improvement in fit was justified by the additional free parameters (Eq 1) [59].
1

Where AICc is the corrected Akaike Information Criteria measure, N is the sample size (113), RSS is the residual sub square of errors for the different models, and K is the number of parameters (2, 5, 8, or 11 for one or the sum of two, three, or four normal distributions, respectively). According to this criterion, three normal populations provided a significantly better fit than one, two, or four populations.

Statistical analysis of differences in mean vales for cell size and action potential properties were determined by t test. Differences in proportions of cells were evaluated using Fisher’s exact test.
